# A controlled evaluation of the effect of social prescribing programs on loneliness for adults in Queensland, Australia (protocol)

**DOI:** 10.1186/s12889-022-13743-3

**Published:** 2022-07-19

**Authors:** G. A. Dingle, L. S. Sharman, S. Hayes, D. Chua, J. R. Baker, C. Haslam, J. Jetten, S. A. Haslam, T. Cruwys, N. McNamara

**Affiliations:** 1 School of Psychology, The University of Queensland, St Lucia QLD, Brisbane, 4072 Australia; 2 Inala Primary Care, Brisbane, Australia; 3 Primary & Community Care Services, Gold Coast, Australia; 4 Australian National University, Canberra, Australia; 5 Nottingham Trent University, Nottingham, UK

**Keywords:** Loneliness, Social prescribing, Social identity, Group programs, Community

## Abstract

**Background:**

In social prescribing, link workers support individuals whose persistent health problems are exacerbated by loneliness by connecting them to community-based social activities. This approach is well established in the UK and is gaining attention in Australia. However, a major limitation of research to date has been a lack of theoretically informed and rigorous evaluations of social prescribing. We will address these points in this study, applying a social identity framework to examine the effects of group-based social prescribing (SP) activity compared to primary care treatment as usual (TAU).

**Methods:**

Ninety participants experiencing loneliness recruited from primary care services and community centres across five sites in Southeast Queensland will be assigned to one of two conditions (SP, TAU) and assessed at two timepoints (baseline, + 8 weeks). Individuals will be aged 18 years and over, have sufficient English language skills to provide consent, and at the time of recruitment they will not be experiencing acute symptoms or social issues that require urgent intervention. Primary outcomes are loneliness, mental well-being, and health service use (total number of GP, hospital, and allied health visits in the past 3 months). Secondary outcomes will assess social group processes, including number of important social groups, new group identification, multiple identity compatibility, and group-based support and emotion regulation.

**Discussion:**

This study will provide comprehensive data about the extent to which, and how, social prescribing to community-based group activities may help people to feel less lonely, more socially integrated, and healthy over the first 8 weeks. If effective, this social identity-informed model of social prescribing can be disseminated in communities across Australia.

**Trial registration:**

ANZCTR, Registered 8 June 2022 - Retrospectively registered, https://www.anzctr.org.au/ACTRN12622000801718.aspx

## Background

Loneliness is a painful emotional state that occurs when people feel that their social needs are not being met [1]. In Australia, surveys indicate that between 12 and 25% of people experience problematic levels of loneliness [2, 3]. It is recognised as a pressing public health issue worldwide [4, 5]. This is because loneliness has serious consequences for individual health and wellbeing, including an increased risk of coronary heart disease and stroke, depression, substance use, and premature death [6–9]. It is also associated with frequent attendance at general (medical) practice appointments and hospital emergency departments [10, 11]. It is important to understand and address loneliness in the context of increasing demand on health services and the need to reduce inappropriate health care usage.

Internationally, there is a growing interest in community-based preventative solutions to loneliness as part of a holistic approach to health care. Among these, social prescribing programs are gaining considerable traction [12]. The UK model of social prescribing is integrated into GP clinics and involves health professionals referring individuals to a link worker who supports them to join social activity groups delivered by diverse facilitators within the community [13–15]. Well-known models of social prescribing include ‘arts on prescription’ [16], ‘education on prescription’ [17], and ‘exercise on prescription’ [18]. A randomised controlled trial [19], single arm trials [20–23], and a systematic review [24] have found that social prescribing programs can be effective in reducing participants’ social isolation and loneliness, increasing community integration, and improving mood and mental well-being. However, there is a large variation in the models of social prescribing and the methods used to evaluate them [25], and this has led some researchers to be critical of the approach [26]. This has prompted calls for a theoretical framework to inform clients, link workers, and referring agents about how social prescribing can be implemented most effectively. The social identity approach has been identified as having particular value for social prescribing [27, 28].

The theoretical framework that forms the basis for the study builds upon social identity theory and self-categorisation theory [29, 30]. Together, these theories account for the role that social group memberships play in people’s lives via integration as part of their sense of self or identity. When people self-categorise as belonging to certain groups (e.g., as members of a national, gender, family, occupational, community, or sports group), they tend to adopt the norms, values, and attitudes of those groups as their own. The stronger their social identification with a group and its members, the more likely it is that they will behave in ways that reflect the beliefs and values of that group [31]. Put simply, it is this sense of identification that gives social groups the power to influence a person’s thoughts, feelings, and behaviour [32]. Furthermore, depending on the group’s norms and values, this influence can be positive (e.g., leading to the provision of support) or negative (e.g., leading to discriminatory behaviour).

Most relevant to the present protocol is the social identity approach to health and well-being, which has become known as the *social cure* approach [33, 34]. According to the social cure approach, groups that we identify with provide us with access to a range of psychological resources — such as social support, an enhanced sense of control, a sense of belonging, self-esteem, and emotion regulation [35–37]. These in turn help people to cope and manage the many challenges they face in health contexts. The more positive group memberships a person has, the more resources they can draw upon as they need. In line with these claims, emerging evidence from social prescribing research in the UK indicates that an increase in the number of groups that people belong to leads to better health-related quality of life [38] and improved health service usage [23]. Moreover, these effects appear to be in line with predictions from the social identity approach, with reductions in loneliness flowing from an increased sense of community belonging and social support.

Research interrogating the social cure approach has contributed to development of the social identity model of identity change (SIMIC) [34, 39], which specifies the group processes that support health and well-being in the context of life change (e.g., transitions to university study, parenthood, recovery from illness). This model argues that life transitions typically entail a loss of social group memberships and related social identities, and that, other things being equal, this will tend to compromise wellbeing. At the same time, though, SIMIC argues that in the context of life transitions, multiple group memberships and associated identities are generally protective of health and wellbeing. This is because having access to multiple group memberships (a) increases people’s ability to maintain some of these in the context of the transition (and thereby have a sense of social identity continuity) and (b) provides them with a platform for developing new group memberships (and thereby have a sense of social identity gain) and should thereby increase people’s access to health-supporting psychosocial resources. This should be more true to the extent that different group memberships are compatible as this makes them easier to manage and less stressful [40, 41].

The social identity approach argues that a lack or loss of group memberships is a significant determinant of loneliness [42]. Moreover, this lack of group memberships is often a particular problem for people who experience economic disadvantage, disability, or chronic health problems [3]. SIMIC suggests that here loneliness can result from the fact that those who are marginalised in this way may have limited existing group memberships and identities and therefore few, if any, psychosocial resources to draw upon in stressful times. Here, we are considering the lonely person’s referral to a social prescribing program to be a transitional period whereby they develop a relationship with a link worker and become engaged in a meaningful group activity. The model predicts that, to the extent that the person identifies with their new group, they will be able to access group-based psychosocial resources. Two psychosocial resources that appear most relevant to the experience of loneliness are social support given and received, and emotional sharing with other members of the group. Support given and received have been shown in previous research to mediate the relationship between group identification and (lower) stress and life satisfaction [43]. Group-based emotion regulation is predicted to decrease individuals’ experience of loneliness — itself a negative emotional state [1]. Indeed, research suggests that emotion regulation plays a key role in the dynamics of loneliness [42, 44].

Thus, the study will test the extent to which joining one or more new groups through social prescribing initiatives (2), and the associated social identity gain, provides participants with opportunities to give and receive support from others, and to regulate their own and others’ emotions (5), which are expected in turn, to decrease loneliness, decrease inappropriate health service use, and increase wellbeing (6). In addition, those individuals who are fortunate to have existing group memberships and identities before joining the social prescribing program are expected to report positive outcomes to the extent that they maintain their former groups and identities (3), and that these are compatible (4) with the new group memberships and identities gained through the social prescribing initiative.

The proposed study responds to recent calls for more research into social prescribing (e.g., in the 2021 QLD Parliamentary Inquiry into social isolation and loneliness, the 2020 Royal Australian College of General Practitioners / Consumers Health Forum Round Table report, and the 2020 Friends for Good More than Medicine report). It also addresses the need for high-quality evidence to evaluate social prescribing in an Australian context. We will evaluate the effectiveness of social prescribing, in comparison to GP treatment as usual (TAU), in reducing loneliness, as well as its effects on wellbeing and health service usage across an 8-week period.

We hypothesise that participants who participate in social prescribing will show significant improvements in loneliness and wellbeing and lower use of health services relative to those in the TAU condition. Second, the study will test the processes through which observed outcomes occur, with a focus on evaluating the two pathways in the expanded SIMIC-based model to determine whether number of important social groups, new group identification, old and new group identity compatibility, and group-based psychological resources (support given and received, and emotion regulation) are implicated in reduced loneliness and health service use and increased wellbeing over the course of the study.

## Methods

### Design and participants

While a randomised controlled design would be the strongest methodology, it requires participants to be interested and available to attend sessions with a link worker and then group activities as part of the social prescribing intervention if they are allocated to the treatment condition. It would be difficult to retain participants in social prescribing who are not interested and/or available, and conversely, it would be unethical to withhold social prescribing from participants who are interested and available, given evidence that it is potentially effective in managing loneliness. Accordingly, as shown in Fig. 2, our study will have a non-randomised controlled (parallel) design, with a social prescribing condition (SP) and a primary care treatment as usual (TAU) condition.

This research will take place in 5 sites in Southeast Queensland where social prescribing is offered, using a combination of GP and community centre models. The sites are in areas with diverse populations in terms of socioeconomic and cultural backgrounds: a community centre in a southern suburb of Brisbane, a GP clinic in the same suburb, a GP clinic in a southwestern suburb of Brisbane, a community centre in a northern suburb of Brisbane, and a combined GP and community centre in a regional city located about an hour’s drive south of Brisbane.

*Social Prescribing participants* (SP) will be adults aged 18 and over, experiencing loneliness or social isolation who are eligible for referral to the social prescribing programs at these sites. Local General Practitioners and Allied Health Professionals expected to be the most likely referral pathway. Recruitment will be promoted through the authors giving talks to referring agents and through project steering meetings. Participants will have sufficient English language skills to be able to provide consent to participate in the research. We expect them to range in age, and to represent a variety of cultural and ethnic groups. Exclusion criteria are that the individual reports acute symptoms (such as suicidal ideation, manic or agitated behaviour, or intoxication), or an acute social issue (such as domestic violence or housing crisis that would need to take priority), that would interfere with their capacity to engage with SP. Such individuals will be directed by the Link Worker to a more suitable service. Link Workers will refer clients who indicate an openness to participating the study to the researchers who will contact individuals to confirm their consent to participate in the study.

*Treatment as Usual participants* (TAU) will be selected from GP clinics in the same locations as the social prescribing programs and will meet our inclusion and exclusion criteria. A staff member within each of these services will identify frequent attending patients who will be contacted by telephone by the researchers and invited to participate in the study. Frequently attending patients will be defined as patients attending the practice 12 or more times each year over a two-year period who have not already been referred to a social prescribing scheme. These parameters remove patients attending frequently for short-lived medical needs, such as a pregnancy. We will assess for baseline differences between the conditions and statistically control for any variables that are found to differ significantly at baseline.

### Power calculation

An a priori power analysis indicates that a sample size of 90 would be required to detect a significant difference between groups with a medium effect size (*f* = .30) on primary outcome variables, with a power of 0.8 and a default alpha of .05, using the planned 2 X 2 mixed analysis of variance. Our goal for recruitment is therefore to continue until we reach 90 with data at both time points (i.e., 45 in each condition).

### Data collection

Data will be collected via survey by researchers either in-person or online when in-person data collection is not feasible. Participants will be allocated a study code so that no identifying information is collected with the surveys. The initial (T1) survey will be collected when participants first engage in social prescribing (i.e., when they begin to meet with the Link Worker, but are yet to attend a group or have only recently started attending a group activity). Participants will then be followed up 8-weeks later (T2). The number of group activities a participant joins during the 8-week period is up to the discretion of the participant and Link Worker, however, we expect that this will typically be just one new group. Surveys will be collected from the TAU participants across the same time interval of 8 weeks, and they will continue to receive primary care treatment as usual. Participants will be reimbursed with AUD $40 vouchers at T1 and T2. Non-identified service audit data will also be collected by researchers from the Link Workers to gain an overall sense of the total number of program referrals and the different referral pathways, as well as general demographic characteristics.

### Measures

***The primary outcomes are loneliness, wellbeing, and health service use, and these will be assessed in both T1 and T2 surveys.***

*Loneliness* will be assessed via the 8-item brief UCLA Loneliness Scale [45] (e.g., ‘how often do you feel left out?’), in which responses are made on a 4-point scale (where 0 = *never*, 1 = *rarely*, 2 = *sometimes*, and 3 = *often*). Items are summed to produce a total score ranging from 0 to 24.

*Wellbeing* will be measured using the 14-item Warwick Edinburgh Mental Wellbeing Scale [46]. The items are positively worded and ask respondents how often they have experienced various psychological states over the past 2 weeks (e.g., ‘I’ve been feeling relaxed’) on a 5-point rating scale (where 1 = *none of the time* to 5 = *all of the time*). Scores are summed to produce a total score in the range from 14 to 70, with higher scores corresponding to a higher level of mental well-being. The WEMWBS is psychometrically robust in general populations, with excellent internal consistency (α = 0.91) and good test-retest reliability (*r* = .83) [46].

*Health Service Use* will be measured by the self-reported total number of visits that participants make to hospital, community mental health services, counsellor, psychologist, psychiatrist, social worker, GP (in person, over the phone, or via home visit) over the course of the study (adapted from the measure used in [47]). In addition, respondents will be asked to rate their current health on a single 5-point scale (where 1 = *very poor* to 5 = *very good)*.

Secondary outcomes are process measures by which the social prescribing intervention is expected to produce effects on the primary outcomes: group memberships and identities, new group identification, former and new group compatibility, and group-based psychosocial resources of social support and emotion regulation.

*Group memberships and identities* Participants will be asked to list up to six groups that they are members of and rate the importance of each on a 7-point scale (where 1 = *not important* to 7 = *very important).* From this, the number of important groups (i.e., those rated 5–7) will be derived (see [34], p. 350–351).

*New group membership and identification* Social identification with the (first) new group that the participant has joined through the social prescribing program will be measured by 4-items rated on a 5-point scale (where 1 = *strongly disagree* to 5 = *strongly agree* [48];).

*Group compatibility* will be measured by averaging 2 items: ‘Being a member of my new group/s is compatible with being a member of my other groups’ and ‘The norms and values of my various groups are compatible with each other’, both rated on a 5-point scale (where 1 = *strongly disagree* to 5 = *strongly agree* [34];, pp. 349–350).

*Group-based psychological resources* are defined as social support received and provided, measured by an average of scores on 2 items from [43]: ‘I get support from people in my new group’ and ‘I provide support to others in my new group’. Each is rated on a 5-point scale (where 1 = *strongly disagree* to 5 = *strongly agree*). Emotion regulation was measured using a 12-item version of the Emotion Regulation of Others and Self measure [49], which has been validated in previous research using a large sample representative of the UK population [42]. In this study, we use the two interpersonal EROS subscales assessed by 3 items each: extrinsic affect improving (e.g., ‘I discussed someone’s positive characteristics to try to improve how they felt’) and extrinsic affect worsening (e.g., ‘I explained to someone how they had hurt myself or others to try to make the person feel worse’).

### Analysis

**Fig. 1:**
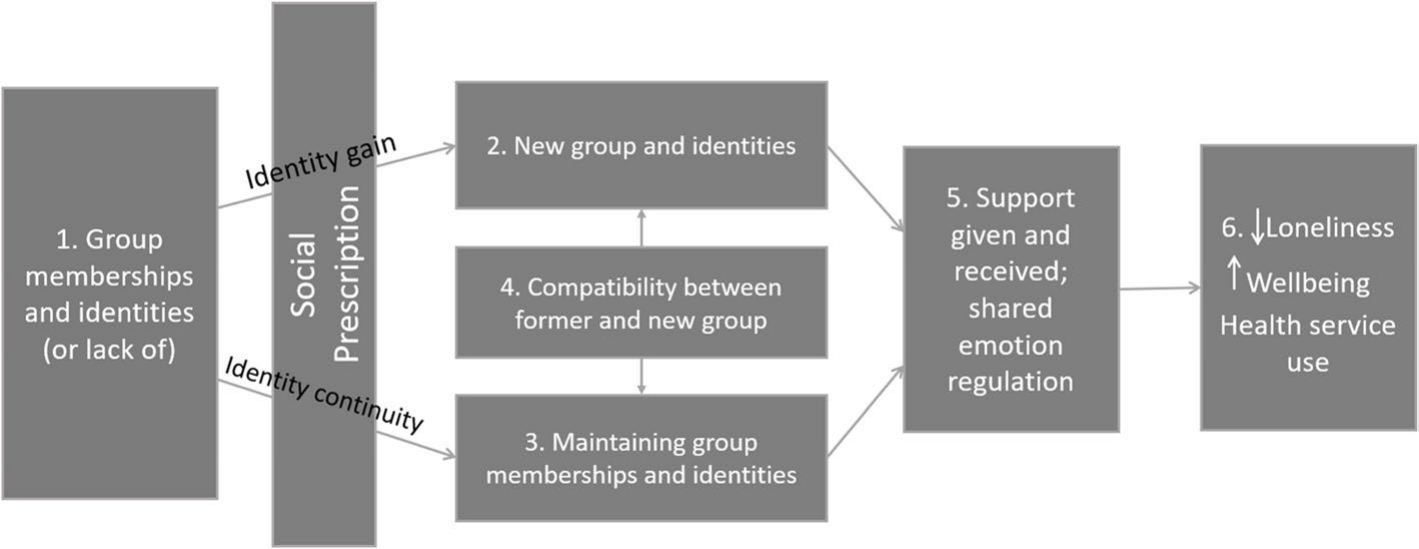
The extended SIMIC-based model of social prescribing that will be evaluated in the study

To assess the effect of the SP intervention, group differences on the primary and secondary outcome measures will be examined using a series of mixed-effects repeated measures (MMRM) models, which will specify timepoint, participant, and condition as levels in the analyses. MMRM is a full-information maximisation likelihood estimation strategy that can model data even when some observations are missing, and thus honours the intention-to-treat principle [50]. For all hypothesis testing, both intention-to-treat analyses (ITT; where all available data are included in MMRM) and per-protocol analyses (PP; only including participants who met eligibility criteria throughout the trial period, completed the intervention according to the protocol and had both baseline and follow up data) will be completed. Furthermore, we will test the extended Social Identity Model of Identity Change (Fig. 1) using a structural equation modelling approach that accounts for nested data (i.e., individuals within group programs) to test relationships across time between group membership, new group identification, old and new group compatibility, help given to others in the group, interpersonal emotion regulation, and the outcomes of loneliness, wellbeing, and health service use.

**Fig. 2:**
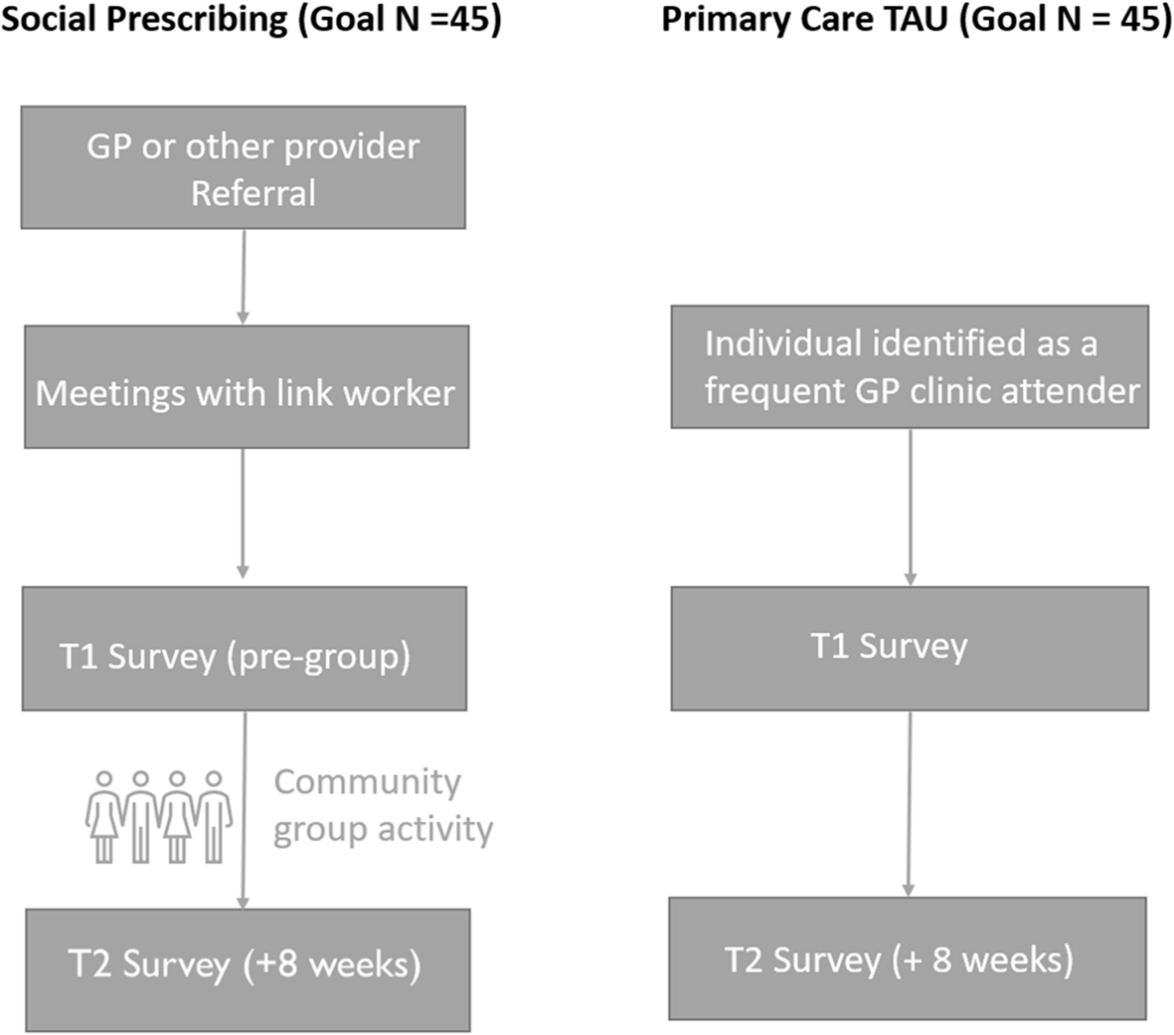
Design for the controlled parallel design study evaluating social prescribing vs GP treatment as usual for loneliness

## Discussion

This project will represent the first controlled evaluation conducted in Australia - a country where such prescribing is only emerging [20, 21]. It will also be one of the few studies that adopts a social cure theoretical framework [33, 34] to understand the processes through which social prescribing to community group activities produces expected changes in loneliness, wellbeing, and health service use (see also [23, 38] in the UK). The wide range of self-report measures and link worker collected service data will provide a rich data set. Specifically, between the two assessments, we expect to find improvements in well-being and reduced loneliness among those engaging with the intervention, and minimal change in these outcomes for participants in the TAU condition.

In line with social cure theorising, and our extended SIMIC-based model of social prescribing, we expect improvements associated with joining a new group activity through social prescribing to be mediated by new group identification, old and new group compatibility, and group based psychological resources which are gained. These changes are expected to contribute to reduced health service use at follow-up, as has been observed in previous research in which adults who frequently attend GP clinics met their social needs through meaningful group participation [10].

The findings will be disseminated via a final report, a summary communicated to the participants and stakeholders by email, and in scholarly publications and presentations. They will inform policy and practice developments in the community and health sectors in Queensland and potentially have impact across Australia and beyond. This research is timely, as there have been growing calls for policy- and evidence-based interventions to address social isolation and loneliness, especially in the wake of the COVID-19 pandemic and related social distancing and lockdowns [51–54]. The absence of high-quality evidence and a rigorous theoretical framework is the primary barrier to wider implementation of a social prescribing approach and this study fills both of those gaps.

## Data Availability

The datasets generated and analysed during the current study will be available in the University of Queensland Data Management System repository, for 5 years after publication on approval of the corresponding author by email: dingle@psy.uq.edu.au.

## Abbreviations

SP: Social prescribing
TAU: Treatment as usual
GP: General Practitioner
SIMIC: Social identity model of identity change
QLD: Queensland
UCLA: University of California, Los Angeles
WEMWBS: Warwick Edinburgh Mental Well-being Scale
EROS: Emotion Regulation of Others and Self measure
MMRM: Mixed-effects repeated measures analysis
ITT: Intention to treat approach to analysis
